# The social life of voices: studying the neural bases for the expression and perception of the self and others during spoken communication

**DOI:** 10.3389/fnhum.2015.00129

**Published:** 2015-03-19

**Authors:** Carolyn McGettigan

**Affiliations:** Department of Psychology, Royal Holloway, University of LondonEgham, UK

**Keywords:** voice, social neuroscience, speech perception, speech production, functional neuroimaging, identity

## Introduction

In 2013, London Underground reinstated the actor Oswald Laurence's famous “Mind the gap” announcement at Embankment station, having learned that the widow of the actor had been regularly visiting this station since her husband's death in order to hear his voice again (Hope, 2013). Even in the absence of a personal connection to the couple, it is easy to find this an emotionally affecting story. Anecdotally, “It's so nice to hear your voice” is commonly encountered in telephone conversations with loved ones, yet there is relatively little known about the cognitive and neural underpinnings of this expression. Similarly, a sense of ownership of one's voice has important implications—companies like VocalID (www.vocalid.co) have recognized the impact of providing individualized voices to patients who rely upon synthesizers for communication—but, to date, the neuroscience of speech production has been predominantly concerned with the accurate formulation of linguistic messages.

Although there are relatively unchanging aspects of every voice, due to the anatomical constraints of the talker's vocal tract as well as body size and shape (Kreiman and Sidtis, 2011), it is also important to note that the voice is not a static object. There is no such thing as a passive voice; the voice (like all sounds) demands an action to occur for it to exist at all (Scott and McGettigan, 2015). Much of our vocal expression is the result of voluntary motor acts, which can be modified consciously in response to changes in acoustic, informational and social demands (McGettigan and Scott, 2014; Scott and McGettigan, 2015). Sidtis and Kreiman (2012) write that the voice is “revelatory of ‘self,’ mental states, and consciousness,” reflecting “both the speaker and the context in which the voice is produced” (p. 150). It is thus a *dynamic self* that is modified according to the talker's goals, affecting both the talker and the addressee in their roles as perceivers and producers of verbal and non-verbal vocal signals.

Disruption to paralinguistic aspects of voice perception and production has implications for psychosocial wellbeing. Most reports of Foreign Accent Syndrome—where patients produce altered speech that perceptually resembles a non-native accent (e.g., due to brain injury, or orofacial surgery)—concentrate on the phonetic, perceptual and neurological correlates of the disorder, yet there is evidence that there can also be significant impacts on the patient's sense of self-identity (Miller et al., 2011; DiLollo et al., 2014). In voice perception, difficulties in the recognition of emotional and attitudinal prosody have implications for effective psychosocial function in healthy aging, schizophrenia, and autism (Mitchell and Ross, 2013). It is thus crucial that neurobiological accounts of speech and voice processing consider not just what is said, but *how it is said*, in order to characterize the human aspects of vocal communication behaviors.

## Listening to spoken selves—the importance of personally familiar voices

An influential early functional MRI study compared the neural responses to the voices of human men, women and children with those to non-human sounds (Belin et al., 2000). This revealed enhanced activation to voices in bilateral regions of the superior temporal cortex, which became known as the “Temporal Voice Areas” (TVAs). Further work exploring the perceptual processing of individual vocal identities has typically implicated right-dominant activation in the anterior superior temporal sulcus (Belin and Zatorre, 2003; von Kriegstein et al., 2003, 2005; Kriegstein and Giraud, 2004; Schall et al., 2015). More recently, temporal activations were associated with the perception of acoustic differences between voices, while purely identity-related responses were found in right inferior frontal cortex (Latinus et al., 2011). Similar profiles of right-dominant temporal activation have been also observed in the perception of acoustic cues in affective vocal signals, with additional engagement of prefrontal cortex and the limbic system (including the amygdala, and dorsolateral and medial prefrontal cortices, depending on specific task demands; see Brueck et al., 2011). However, the neuroscience of voices and emotion has not yet considered *the emotional consequences of hearing other vocal identities*, in particular those of highly familiar and valued others.

Presumably due to methodological constraints, the majority of work on paralinguistic voice processing has involved the perception of unfamiliar or newly learned vocal identities. This overlooks the social and emotional salience associated with hearing the voices of trusted friends and loved ones. Sidtis and Kreiman (2012) write: “Personally relevant voices, by definition, are represented in memory with emotional reference to the self” (p. 154). Mechanistically, Kreiman and Sidtis (2011) suggest that unfamiliar voice perception is based on distinguishing local acoustic features, whereas identification of familiar voices involves comparing a heard stimulus to representations in long-term memory, and this dissociation is supported by neuropsychological evidence from cases of phonagnosia (Van Lancker et al., 1988; Garrido et al., 2009). There are also implications from neuroimaging studies that known voices engage higher order responses that could reflect their social salience. For example, personally familiar voices have engaged responses in anterior regions of the temporal lobe, the precuneus and frontal poles (e.g., Nakamura et al., 2001; Shah et al., 2001), which in other literatures have been associated with autobiographical memory and the “social brain” (concerned with the processing of mental states and intentions in others; Blakemore, 2008). To date, however, the use of sets of “commonly familiar” voices including a mix of friends, colleagues, relatives or celebrities has enabled the identification of overall responses to familiarity in vocal signals, but has limited the investigation of the higher-order meaning of those individual voices as social signals for the listener (Sugiura, 2014). Thus, to literature on familiar voice processing has so far offered no clues as to the neural basis for the significance of voices as described in the London Underground story above.

Therefore, there are remaining questions about how familiar voices of different types—family members, friends, colleagues, romantic partners—engage the brain during the perception of vocal signals. A number of existing studies have shown evidence for heightened release of oxytocin—a hormone associated with parental and interpersonal bonding—when participants hear the voice of a loved one (Seltzer et al., 2010). Seltzer and colleagues additionally showed that vocal communication between mothers and daughters is more effective at reducing blood cortisol levels (a marker of stress) than text communication (Seltzer et al., 2012). Abrams and colleagues (Abrams et al., 2013) showed evidence for reduced structural and functional connectivity between posterior temporal regions associated with speech perception and the brain's reward systems (involving ventral tegmental area (VTA), nucleus accumbens, left insula, orbitofrontal cortex, ventromedial prefrontal cortex) in children with autism, suggesting that this may be related to this group's relative lack of engagement with spoken signals in day-to-day interactions. There is, however, no detailed account of the behavioral correlates of this functional interaction of speech and reward systems in typical populations. To understand the voice as a social signal, it is essential to investigate the interplay between linguistic and non-linguistic (affective, reward, social) networks during vocal communication. To date, the considerable methodological demands of obtaining controlled, participant-specific vocal recordings of personally valued others has precluded such an investigation (Sidtis and Kreiman, 2012).

## Producing the self voice—speaking in a social context

Recent neurobiological models of speech production have adopted a forward models approach, in which the brain aims to reduce the error between the predicted and actual sensory consequences of a spoken utterance (Guenther, 2006; Hickok et al., 2011; Guenther and Vladusich, 2012; Hickok, 2012). Here, the goal is the accurate delivery of spoken language at the phonemic and syllabic level. Although what we say (i.e., the choice of words) is important for informational and social exchanges, so too is the way we say it. In vocal communication, we adopt a variety of “selves,” which we use flexibly to achieve the social and informational goals of the conversation, even if the linguistic message itself remains constant across contexts (McGettigan et al., 2013; Hughes et al., 2014)—consider the tone of voice used with a close family member vs. that used with colleagues at work. We carried out the first functional imaging study of this voluntary modulation of the spoken self, using an impersonations task (McGettigan et al., 2013). We found evidence engagement of the left anterior insula and the frontal operculum in the modulation of vocal identity during spoken sentence production, with stronger interaction of these regions with right-dominant superior temporal voice perception areas supporting the emulation of specific target identities. A similar study asking participants to voluntarily introduce a phonological (prosodic or segmental) modification during the repetition of heard speech engaged left-dominant activations in inferior frontal and inferior parietal cortex (Peschke et al., 2012). Future developments to neurobiological models of speech production should incorporate these paralinguistic aspects of self-expression, for example taking into account the possibility that the auditory and somatosensory targets of speech production may be adjusted systematically depending on the talker's mood, communicative intentions and their personal relationship to the intended audience.

An improved understanding of how flexible control of the voice affords the attainment of social goals demands investigation of how the talker's intentions are expressed in speech, detected by the listener and used to elicit or guide further social behaviors between interlocutors. *Phonetic convergence* describes the phenomenon of interlocutors aligning their acoustic-phonetic pronunciation of speech over a period of spoken interaction, often outside of their conscious awareness (Krauss and Pardo, 2006). Pardo and colleagues have measured convergence in a variety of settings, including contexts where talkers work toward a shared goal (e.g., in a map-reading task; Pardo, 2006; Pardo et al., 2013). This convergence is correlated with interpersonal liking—Pardo et al. (2012) found that the degree of phonetic convergence between pairs of college roommates was moderately related to their self-reported social closeness. Further, Adank et al. (2013) found evidence for a causal association between imitation and social processing, where overt imitation of a talker led to increased ratings of the social attractiveness of that voice. Neuroimaging studies investigating participants' phonetic convergence with recorded speech targets have found activations in bilateral auditory cortex and inferior parietal cortex associated with conscious and unconscious aspects of the phenomenon (Peschke et al., 2009; Sato et al., 2013). However, measurable evidence for phonetic convergence is highly variable, across participants and social contexts, and it can even be the case that convergence on one feature can be accompanied by divergence on another within the same cohort (Pardo, 2010, 2013). This may be due to issues associated with measurement selection, the fidelity of the talker's phonetic perception of the other interlocutor and the situational context. The challenge for future research in this area is to identify the mechanisms underlying convergence and its social consequences in a way that can cope with this variability in behavior.

## Future directions for the neuroscience of vocal communication

Pardo (2012) writes: “Talkers speak to be understood, and understanding means more than intelligibility” (p. 764). I suggest that this should act as a starting point for the onward development of the neuroscience of human vocal behavior, and propose the following considerations for future work in the area:

- Studies of vocal identity perception should make more regular and selective use of familiar voices, in order to interrogate the interaction of speech/voice perception systems with other response networks relevant to social interactions. It is important to consider that there are different types of familiar person, for whom the perceptual response may systematically differ. Alternatively, Sugiura (2014) suggests that more tightly controlled investigation of the social significance of familiar others could be achieved experimentally by training participants to associate particular social attributes with virtual agents.
- Studies of speech production mechanisms should consider the intended recipient of the spoken message and their relationship to the talker. Here, neuroimaging techniques may offer a means of investigating the interaction of speech perception and production systems with affective, reward and motivational responses, in both the presence and absence of measurable behavioral changes in the phonetic realization of speech.
- The advent of improved methodological approaches to brain imaging during speech production (e.g., Xu et al., 2014) presents mounting pressure to examine vocal behavior in its most typical context: conversation. Garrod and Pickering (2004, 2009) advocate this approach in their Interactive Alignment model of dialog, where it is proposed that conversation proceeds smoothly via the alignment of the interlocutors' mental models. A recent investigation of brain-to-brain correlations during storytelling has identified significant and extensive coupling between the producer and the comprehender in regions previously associated with higher-order mentalizing and theory of mind tasks (Silbert et al., 2014). Application of such approaches using dyads varying in the type and quality of the interlocutors' relationship could form a promising avenue to investigate how speech networks interact with social and affective networks during exchanges with familiar others.

### Conflict of interest statement

The author declares that the research was conducted in the absence of any commercial or financial relationships that could be construed as a potential conflict of interest.

## References

[B1] Abrams D. A. Lynch C. J. Cheng K. M. Phillips J. Supekar K. Ryali S.. (2013). Underconnectivity between voice-selective cortex and reward circuitry in children with autism. Proc. Natl. Acad. Sci. U.S.A. 110, 12060–12065. 10.1073/pnas.1302982110 23776244 PMC3718181

[B2] Adank P. Stewart A. J. Connell L. Wood J. (2013). Accent imitation positively affects language attitudes. Front. Psychol. 4:280. 10.3389/fpsyg.2013.00280 23734137 PMC3659325

[B3] Belin P. Zatorre R. J. (2003). Adaptation to speaker's voice in right anterior temporal lobe. Neuroreport 14, 2105–2109. 10.1097/00001756-200311140-00019 14600506

[B4] Belin P. Zatorre R. J. Lafaille P. Ahad P. Pike B. (2000). Voice-selective areas in human auditory cortex. Nature 403, 309–312. 10.1038/35002078 10659849

[B5] Blakemore S.-J. (2008). The social brain in adolescence. Nat. Rev. Neurosci. 9, 267–277. 10.1038/nrn2353 18354399

[B6] Brueck C. Kreifelts B. Wildgruber D. (2011). Emotional voices in context: a neurobiological model of multimodal affective information processing. Phys. Life Rev. 8, 383–403. 10.1016/j.plrev.2011.10.002 22035772

[B7] DiLollo A. Scherz J. Neimeyer R. A. (2014). Psychosocial implications of foreign accent syndrome: two case examples. J. Constr. Psychol. 27, 14–30 10.1080/10720537.2013.819305

[B8] Garrido L. Eisner F. McGettigan C. Stewart L. Sauter D. Hanley J. R.. (2009). Developmental phonagnosia: a selective deficit of vocal identity recognition. Neuropsychologia 47, 123–131. 10.1016/j.neuropsychologia.2008.08.003 18765243

[B9] Garrod S. Pickering M. J. (2004). Why is conversation so easy? Trends Cogn. Sci. 8, 8–11. 10.1016/j.tics.2003.10.016 14697397

[B10] Garrod S. Pickering M. J. (2009). Joint action, interactive alignment, and dialog. Top. Cogn. Sci. 1, 292–304. 10.1111/j.1756-8765.2009.01020.x 25164934

[B11] Guenther F. H. (2006). Cortical interactions underlying the production of speech sounds. J. Commun. Disord. 39, 350–365. 10.1016/j.jcomdis.2006.06.013 16887139

[B12] Guenther F. H. Vladusich T. (2012). A neural theory of speech acquisition and production. J. Neurolinguistics 25, 408–422. 10.1016/j.jneuroling.2009.08.006 22711978 PMC3375605

[B13] Hickok G. (2012). Computational neuroanatomy of speech production. Nat. Rev. Neurosci. 13, 135–145. 10.1038/nrn3158 22218206 PMC5367153

[B14] Hickok G. Houde J. Rong F. (2011). Sensorimotor integration in speech processing: computational basis and neural organization. Neuron 69, 407–422. 10.1016/j.neuron.2011.01.019 21315253 PMC3057382

[B15] Hope C. (2013). ‘Mind the Gap’ Voice Restored to London Underground After Widow Missed her Husband's Voice, The Telegraph. Available online at: http://www.telegraph.co.uk/finance/newsbysector/transport/9918662/Mind-the-Gap-voice-restored-to-London-Underground-after-widow-missed-her-husbands-voice.html

[B16] Hughes S. M. Mogilski J. K. Harrison M. A. (2014). The perception and parameters of intentional voice manipulation. J. Nonverbal Behav. 38, 107–127 10.1007/s10919-013-0163-z

[B17] Krauss R. M. Pardo J. S. (2006). Speaker perception and social behavior: bridging social psychologyand speech science, in Bridging Social Psychology: The Benefits of Transdisciplinary Approaches, ed van Lange P. A. M. (Hillsdale, NJ: Erlbaum), 273–278.

[B18] Kreiman J. Sidtis D. (2011). Foundations of Voice Studies: An Interdisciplinary Approach to Voice Production and Perception. Hoboken, NJ: Wiley-Blackwell.

[B19] Kriegstein K. V. Giraud A. L. (2004). Distinct functional substrates along the right superior temporal sulcus for the processing of voices. Neuroimage 22, 948–955. 10.1016/j.neuroimage.2004.02.020 15193626

[B20] Latinus M. Crabbe F. Belin P. (2011). Learning-induced changes in the cerebral processing of voice identity. Cereb. Cortex 21, 2820–2828. 10.1093/cercor/bhr077 21531779

[B21] McGettigan C. Eisner F. Agnew Z. K. Manly T. Wisbey D. Scott S. K. (2013). T'ain't what you say, it's the way that you say it—left insula and inferior frontal cortex work in interaction with superior temporal regions to control the performance of vocal impersonations. J. Cogn. Neurosci. 25, 1875–1886. 10.1162/jocn_a_00427 23691984 PMC4050359

[B22] McGettigan C. Scott S. K. (2014). Voluntary and involuntary processes affect the production of verbal and non-verbal signals by the human voice. Behav. Brain Sci. 37, 564–565. 10.1017/S0140525X13004123 25514954

[B23] Miller N. Taylor J. Howe C. Read J. (2011). Living with foreign accent syndrome: insider perspectives. Aphasiology 25, 1053–1068 10.1080/02687038.2011.573857

[B24] Mitchell R. L. C. Ross E. D. (2013). Attitudinal prosody: what we know and directions for future study. Neurosci. Biobehav. Rev. 37, 471–479. 10.1016/j.neubiorev.2013.01.027 23384530

[B25] Nakamura K. Kawashima R. Sugiura M. Kato T. Nakamura A. Hatano K.. (2001). Neural substrates for recognition of familiar voices: a PET study. Neuropsychologia 39, 1047–1054. 10.1016/S0028-3932(01)00037-9 11440757

[B26] Pardo J. S. (2006). On phonetic convergence during conversational interaction. J. Acoust. Soc. Am. 119, 2382–2393. 10.1121/1.2178720 16642851

[B27] Pardo J. S. (2010). Expressing oneself in conversational interaction, in Expressing Oneself/Expressing One's Self: A Festschrift in Honor of Robert M. Krauss, ed Morsella E. (London: Taylor and Francis), 183–196.

[B28] Pardo J. S. (2012). Reflections on phonetic convergence: speech perception does not mirror speech production. Lang. Linguistics Compass 6, 753–767 10.1002/lnc3.367

[B29] Pardo J. S. (2013). Measuring phonetic convergence in speech production. Front. Psychol. 4:559. 10.3389/fpsyg.2013.00559 23986738 PMC3753450

[B30] Pardo J. S. Gibbons R. Suppes A. Krauss R. M. (2012). Phonetic convergence in college roommates. J. Phon. 40, 190–197 10.1016/j.wocn.2011.10.001

[B31] Pardo J. S. Jay I. C. Hoshino R. Hasbun S. M. Sowemimo-Coker C. Krauss R. M. (2013). Influence of role-switching on phonetic convergence in conversation. Discourse Process. 50, 276–300 10.1080/0163853X.2013.778168

[B32] Peschke C. Ziegler W. Eisenberger J. Baumgaertner A. (2012). Phonological manipulation between speech perception and production activates a parieto-frontal circuit. Neuroimage 59, 788–799. 10.1016/j.neuroimage.2011.07.025 21787870

[B33] Peschke C. Ziegler W. Kappes J. Baumgaertner A. (2009). Auditory-motor integration during fast repetition: the neuronal correlates of shadowing. Neuroimage 47, 392–402. 10.1016/j.neuroimage.2009.03.061 19345269

[B34] Sato M. Grabski K. Garnier M. Granjon L. Schwartz J.-L. Nguyen N. (2013). Converging toward a common speech code: imitative and perceptuo-motor recalibration processes in speech production. Front. Psychol. 4:422. 10.3389/fpsyg.2013.00422 23874316 PMC3708162

[B35] Schall S. Kiebel S. J. Maess B. von Kriegstein K. (2015). Voice identity recognition: functional division of the right superior temporal sulcus and its behavioral relevance. J. Cogn. Neurosci. 27, 280–291. 10.1162/jocn_a_00707 25170793

[B36] Scott S. K. McGettigan C. (2015). The Voice APA Handbook of Nonverbal Communication. Washington, DC: American Psychological Association.

[B37] Seltzer L. J. Prososki A. R. Ziegler T. E. Pollak S. D. (2012). Instant messages vs. speech: hormones and why we still need to hear each other. Evol. Hum. Behav. 33, 42–45. 10.1016/j.evolhumbehav.2011.05.004 22337755 PMC3277914

[B38] Seltzer L. J. Ziegler T. E. Pollak S. D. (2010). Social vocalizations can release oxytocin in humans. Proc. R. Soc. B Biol. Sci. 277, 2661–2666. 10.1098/rspb.2010.0567 20462908 PMC2982050

[B39] Shah N. J. Marshall J. C. Zafiris O. Schwab A. Zilles K. Markowitsch H. J.. (2001). The neural correlates of person familiarity—a functional magnetic resonance imaging study with clinical implications. Brain 124, 804–815. 10.1093/brain/124.4.804 11287379

[B40] Sidtis D. Kreiman J. (2012). In the beginning was the familiar voice: personally familiar voices in the evolutionary and contemporary biology of communication. Integr. Psychol. Behav. Sci. 46, 146–159. 10.1007/s12124-011-9177-4 21710374 PMC3224673

[B41] Silbert L. Honey C. Simony E. Poeppel D. Hasson U. (2014). Coupled neural systems underlie the production and comprehension of naturalistic narrative speech. Proc. Natl. Acad. Sci. U.S.A. 111, E4687–E4696. 10.1073/pnas.1323812111 25267658 PMC4217461

[B42] Sugiura M. (2014). Neuroimaging studies on recognition of personally familiar people. Front. Biosci. 19, 672–686. 10.2741/4235 24389212

[B43] Van Lancker D. R. Cummings J. L. Kreiman J. Dobkin B. H. (1988). Phonagnosia—a dissociation between familiar and unfamiliar voices. Cortex 24, 195–209. 10.1016/S0010-9452(88)80029-7 3416603

[B44] von Kriegstein K. Eger E. Kleinschmidt A. Giraud A. L. (2003). Modulation of neural responses to speech by directing attention to voices or verbal content. Cogn. Brain Res. 17, 48–55. 10.1016/S0926-6410(03)00079-X 12763191

[B45] von Kriegstein K. Kleinschmidt A. Sterzer P. Giraud A. L. (2005). Interaction of face and voice areas during speaker recognition. J. Cogn. Neurosci. 17, 367–376. 10.1162/0898929053279577 15813998

[B46] Xu Y. S. Tong Y. X. Liu S. Y. Chow H. AbdulSabur N. Y. Mattay G. S.. (2014). Denoising the speaking brain: toward a robust technique for correcting artifact-contaminated fMRI data under severe motion. Neuroimage 103, 33–47. 10.1016/j.neuroimage.2014.09.013 25225001 PMC4312243

